# Contraceptives as possible risk factors for postpartum depression: A retrospective study of the food and drug administration adverse event reporting system, 2004–2015

**DOI:** 10.1002/nop2.121

**Published:** 2018-01-17

**Authors:** Megumi Horibe, Yuuki Hane, Junko Abe, Toshinobu Matsui, Yamato Kato, Natsumi Ueda, Sayaka Sasaoka, Yumi Motooka, Haruna Hatahira, Shiori Hasegawa, Yasutomi Kinosada, Hideaki Hara, Mitsuhiro Nakamura

**Affiliations:** ^1^ Department of Nursing School of Health Sciences Asahi University Gifu Japan; ^2^ Molecular Pharmacology Department of Biofunctional Evaluation Gifu Pharmaceutical University Gifu Japan; ^3^ Laboratory of Drug Informatics Gifu Pharmaceutical University Gifu Japan; ^4^ Medical Database Co., LTD Tokyo Japan; ^5^ United Graduate School of Drug Discovery and Medical Information Sciences Gifu University Graduate School of Medicine Gifu Japan; ^6^ Present address: Department of Environmental Affairs and Citizen Support Gifu Prefectural Government Gifu Japan; ^7^ Present address: Division of Pharmacy Ehime University Hospital Shitsukawa, Toon Ehime Japan

**Keywords:** adverse event reporting system, contraception, FAERS, intrauterine device, nurses, nursing, postpartum depression

## Abstract

**Aim:**

Postpartum depression is a mood disorder that commonly affects women during the early postpartum period. The objective of this study was to analyse the association of postpartum depression with drugs (including contraceptive devices and implants) with spontaneously reported adverse events reported in the US Food and Drug Administration Adverse Event Reporting System database.

**Design:**

Retrospective study.

**Method:**

Reports of postpartum depression events between 2004–2015 were analysed with a reporting odds ratio (ROR) algorithm. The Medical Dictionary for Regulatory Activities was used to identify postpartum depression.

**Results:**

The reporting odds ratios (95% confidence intervals, CI) of levonorgestrel (an intrauterine device with progestogen), etonogestrel (a hormonal contraceptive implant), sertraline and drospirenone (an oral contraceptive) were 12.5 (8.7–18.0), 14.0 (8.5–22.8), 12.2 (6.5–23.1) and 5.4 (2.7–10.9) respectively. Among the drugs in the US Food and Drug Administration Adverse Event Reporting System database, the use of contraceptives or an intrauterine device with progestogen might convey risk for postpartum depression.

## 1 BACKGROUND

Maternity blues is a common mood disorder that affects women during the early postpartum period (FDA's Office of Women's Health). Maternity blues is a non‐psychotic depressive state and is characterized by transient mood swings. Mothers with maternity blues experience feelings of sadness, anxiety, exhaustion and crying that typically begin in the first few days after delivery and may last for up to 2 weeks (Kennerley & Gath, 1989). If symptoms persist beyond this period, it indicates a mood disorder called postpartum depression (PPD).

According to the Diagnostic and Statistical Manual of Mental Disorders, Fifth Edition (DSM‐5; American Psychiatric Association) (American Psychiatric Association, 1998), the criteria for a major depressive episode are as follows: (i) at least five of the following nine symptoms in the same 2‐week period: depressed mood, loss of interest or pleasure, change in weight or appetite, insomnia or hypersomnia, psychomotor retardation or agitation, loss of energy or fatigue, feeling worthlessness or guilt, impaired concentration or indecisiveness, recurrent thoughts of death and/or suicidal ideation or attempt; (ii) these symptoms cause significant distress or impairment; (iii) the episode is not attributable to substance abuse or a medical condition; (iv) the episode is not better explained by a psychotic disorder and (v) the patient has never experienced a manic or hypomanic episode. PPD refers to the development of a depressive illness following childbirth and is a usually unipolar illness, but may comprise part of a bipolar condition. In addition to its manifestation as severe, persistent maternity blues that often occurs shortly postpartum, PPD can occur at any time during the first year after delivery and lasts for a few weeks or months (O'Hara & Swain, 1996). PPD is not classified as a separate disease, but rather is diagnosed as an affective or mood disorders according to the DSM‐5.

Estimates of the incidence of PPD vary. According to the Cochrane review, the rate of PPD is between 3–25% in the first year following delivery (Dennis & Creedy, 2004) and approximately 10% and 15% of women of childbearing age experience PPD (World Health Organization, 2008). According to a meta‐analysis, the average prevalence rate of non‐psychotic PPD is 13% (O'Hara & Swain, 1996).

Previous studies have showed risk factors for PPD, including: (i) depression or anxiety during pregnancy; (ii) biological changes in hormone levels and the age of mother; (iii) chronic health problems and antenatal depression; (iv) psychological stress, lack of social support from friends and relatives and a stressful life; (v) obstetric/paediatrics factors, including unwanted pregnancy and history of loss of pregnancy; and (vi) socioeconomic status (Beck, 2001; Mehta & Mehta, 2014).

PPD is a serious health problem that affects not only mothers but also the health of their children as well (Hipwell, Goossens, Melhuish, & Kumar, 2000). An association between hormonal medication and PPD is widely suspected, but most of the publications relating to this issue are case reports or small observational studies (Duke, Sibbritt, & Young, 2007; Luukkainen, Pakarinen, & Toivonen, 2001; O'Connell, Davis, & Kerns, 2007). The lack of systematic studies on the relationship between hormonal medication, including intrauterine device (IUD) and PPD, indicates that the clinical implications of this relationship are yet to be fully elucidated. PPD is a critical public health problem that can impair maternal–infant interactions (Klainin & Arthur, 2009) and even lead to maternal suicide and infanticide (O'Hara & Swain, 1996). Nurses have an important responsibility to monitor new mothers and give support and continuous care. More information about the connection between drugs and PPD will help nurses give the best possible care to female patients.

Reports of adverse events in clinical practice that are thought to be potentially related to therapies are spontaneously reported by healthcare practitioners and patients to pharmaceutical manufacturers or directly to the US Food and Drug Administration (FDA) (Bates & Evans, 2009). Case reports are summarized in the spontaneous reporting system (SRS) of the FDA Adverse Event Reporting System (FAERS) database. The FAERS database files are publicly available on the FDA website (https://open.fda.gov/data/faers/). The FAERS database is the largest and best‐known adverse event database worldwide; therefore, the FDA uses it for pharmacovigilance activities, such as investigating safety concerns related to a drug.

In this study, we performed a retrospective analysis of PPD events recorded in the FAERS database. This is the first report where the possible relationship between drugs, including hormonal contraceptive implants and PPD, was evaluated by using the data available in the FAERS database analysed using the reporting odds ratio (ROR).

## 2 METHODS

The FAERS dataset was downloaded from the FDA website (U.S. Food and Drug Administration, http://www.fda.gov/Drugs/GuidanceComplianceRegulatoryInformation/Surveillance/AdverseDrug Effects/default.htm, cited 13 June 2016). The FAERS database consists of seven data tables: patient demographic and administrative information (DEMO), drug/biologic information (DRUG), adverse events (REAC), patient outcomes (OUTC), report sources (RPSR), drug therapy start and end dates (THER) and indications for use/diagnosis (INDI). Each table contains information on adverse events submitted to the FDA (http://www.fda.gov/Drugs/GuidanceComplianceRegulatoryInformation/Surveillance/AdverseDrugEffects/). The informatic structure of the FAERS database adheres to the international safety reporting guidelines issued by the International Council for Harmonisation (ICH E2B) (International Council for Harmonisation, 1998). We built our database from the FAERS dataset using FileMaker Pro 13 software (FileMaker, Inc.), according to ASCII Entity Relationship Diagram (ERD) published by the FDA, the Center for Drug Evaluation and Research (CDER) and the Office of Surveillance and Epidemiology (OSE) (U. S. Food and Drug Administration, “ASC_NTS.DOC”).

Adverse events were coded with terms in the Medical Dictionary for Regulatory Activities (MedDRA, http://www.meddra.org/, cited 12 Jan 2017), which is the terminology dictionary used in the FAERS database (ICH, Introductory Guide MedDRA Version 19.0) (International Council for Harmonisation, 2016). Following the FDA's recommendation, we excluded duplicate reports of the same patient from different reporting sources from the analysis and extracted reports (U. S. Food and Drug Administration, “README.DOC”). We extracted reports of PPD using the preferred term (PT) (PT10056393 in the MedDRA version 19.0). Drugs in FAERS are classified into four categories: primary suspect drug (PS), secondary suspect drug (SS), concomitant (C) and interacting (I), according to the anticipated degree of involvement for adverse events. Only reports with the drug code of PS were included in this analysis.

**Figure 1:**
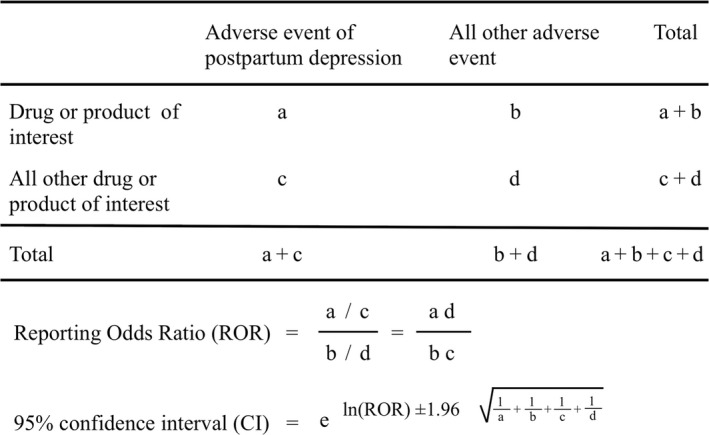
Two‐by‐two contingency table for analysis

To detect PPD incidences, we calculated the ROR, which is established in pharmacovigilance by using a disproportionality analysis (Bates & Evans, 2009). To compare one of the index groups with the reference group, we calculated the crude ROR as *(a/c)/(b/d)* (Figure 1). ROR was expressed as point estimates with a 95% confidence interval (CI). For signal detection, general qualitative judgments are viable; whether a signal is detected or not depends on whether the signal indices exceed predefined thresholds: ROR values <1 indicate no potential exposure–event associations and estimates >1 indicate potential exposure–event safety signals. Safety signals were considered significant when the ROR estimates and the lower limits of the corresponding 95% CI were greater than 1. Two or more cases were required to define the signal (Bates & Evans, 2009). Data analyses were performed using the JMP 11.0 (SAS Institute Inc., Cary, NC, USA).

## 3 RESULTS

The FAERS database contains 7,561,254 reports from January 2004 –December 2015. After excluding duplicates according to the FDA's recommendation, 6,157,897 reports were analysed. The number of reports associated with PPD was 253. Cases of PPD were reported from 17 countries, although 72.8% were from the United States (USA). The reporting ratios for <20, 20–29, 30–39, 40–50 and >50 years old were 5.1%, 50.6%, 38.0%, 4.4% and 1.9% respectively.

The top 10 drugs reported as PS drugs in PPD are summarized in Table 1. The number of reports for the top drugs from highest to lowest is levonorgestrel (IUD with progestogen) = 34, etonogestrel (hormonal contraceptive) = 17, adalimumab (a tumour necrosis factor blocker for the treatment of rheumatoid arthritis) = 13, sertraline (selective serotonin reuptake inhibitor, SSRI) = 10, drospirenone (oral contraceptive) = 8, fluoxetine (SSRI) = 8, topiramate (anticonvulsant) = 8, interferon beta‐1a = 7, quetiapine (atypical antipsychotic) = 7 and tretinoin (retinoic acid) = 5. We evaluated how the suspected drugs were used. Adalimumab was used for rheumatoid arthritis (38%), Crohn's disease (31%), psoriasis (23%) and ulcerative colitis (8%). Interferon beta‐1a was used for multiple sclerosis (71%) and unknown cases (29%). Quetiapine was used for sleep disorder, psychotic disorder, psychotic behaviour and prophylaxis and the reason for use in three cases remains unknown. Tretinoin was used for acne (60%) and some unknown cases (40%). The RORs (95% CI) of levonorgestrel, etonogestrel, sertraline, drospirenone, fluoxetine and topiramate were 12.5 (8.7–18.0), 14.0 (8.5–22.8), 12.2 (6.5–23.1), 5.4 (2.7–10.9), 15.9 (7.9–32.2) and 18.5 (9.1–37.3) respectively. The lower limit of the 95% CI for the RORs exceeded 1, except for adalimumab and interferon beta‐1a (for the treatment of multiple sclerosis).

**Table 1:** The number of reports, reporting odds ratio (ROR) associated with postpartum depression

Drug name	Cases^a^	Total^a^	ROR(95% confidence interval)
Total	253	61,57,897	
Levonorgestrel	34	75,500	12.5 (8.7–18.0)
Etonogestrel	17	31,625	14.0 (8.5–22.8)
Adalimumab	13	2,38,491	1.3 (0.8–2.3)
Sertraline	10	20,628	12.2 (6.5–23.1)
Drospirenone	8	37,105	5.4 (2.7–10.9)
Fluoxetine	8	12,614	15.9 (7.9–32.2)
Topiramate	8	10,881	18.5 (9.1–37.3)
Interferon beta‐1a	7	1,10,957	1.6 (0.7–3.3)
Quetiapine	7	43,888	4.0 (1.9–8.4)
Tretinoin	5	15,763	7.9 (3.2–19.0)

a primary suspect drug (PS).

To evaluate the effect of age on PPD, the reports were stratified into two age groups: less than 30 and over 30 years (Table 2). The number of reports associated with PPD in patients aged <30 or ≥30 years were 88 and 70 respectively. In those aged <30 years, the five most reported drugs were levonorgestrel, etonogestrel, sertraline, acetaminophen and venlafaxine (serotonin and norepinephrine reuptake inhibitor, SNRI). In those aged ≥30 years, the five most reported drugs were levonorgestrel, quetiapine, adalimumab, fluoxetine and citalopram (SSRI).

**Table 2:** Top 10 drugs associated with postpartum depression stratified by age

Drug Name	Number of reports with postpartum depression
<30	88
Levonorgestrel	14
Etonogestrel	10
Sertraline	10
Acetaminophen	9
Venlafaxine	8
Drospirenone	7
Folic Acid	7
Quetiapine	7
Ibuprofen	6
Escitalopram	5
≧30	70
Levonorgestrel	12
Quetiapine	7
Adalimumab	6
Fluoxetine	5
Citalopram	4
Clonazepam	4
Escitalopram	4
Folic Acid	4
Lamotrigine	4
Sertraline	3

We then determined the numbers of the most commonly reported adverse events for the three contraceptive products identified as possible risk factors for PPD in our study: levonogestrel, etonogestrel and drospirenone. For levonogestrel, the top five adverse events reported were device expulsion (20,637), device dislocation (8,374), intrauterine contraceptive device expelled (5,590), pain (5,330), vaginal haemorrhage (5,129) and PPD (34) (Table 3). For etonogestrel, the top five adverse events reported were no adverse event (4,177), pulmonary embolism (3,709), device difficult to use (3,387), product quality issue (2,619), deep vein thrombosis (2,566) and PPD (17). For drospirenone, the top five adverse events reported were pain (12,518), injury (10,470), pulmonary embolism (8,643), deep vein thrombosis (7,921), anxiety (6,557) and PPD (8).

**Table 3:** Number of reports for top 10 of adverse event associated with levonogestrel, etonogestrel and drospirenone

Adverse event	Number of reports
Levonorgestrel (total)	75,500
Device expulsion	20,637
Device dislocation	8,374
Intrauterine contraceptive device expelled	5,590
Pain	5,330
Vaginal haemorrhage	5,129
Abdominal pain	5,091
Uterine perforation	4,868
Genital haemorrhage	4,586
Injury	4,381
Abdominal pain lower	4,087
Postpartum depression^a^	34
Etonogestrel (total)	31,625
No adverse event	4,177
Pulmonary embolism	3,709
Device difficult to use	3,387
Product quality issue	2,619
Deep vein thrombosis	2,566
Medical device complication	2,258
Device breakage	2,018
Metrorrhagia	1,986
Menorrhagia	1,679
Headache	1,625
Postpartum depression^b^	17
Drospirenone (total)	37,105
Pain	12,518
Injury	10,470
Pulmonary embolism	8,643
Deep vein thrombosis	7,921
Anxiety	6,557
Emotional distress	6,234
Cholecystitis chronic	5,683
Cholelithiasis	4,097
Gallbladder disorder	4,037
General physical health deterioration	3,187
Postpartum depression^c^	8

a Postpartum depression rank 359^th^ in terms of levonogestrel.

b Postpartum depression rank 580^th^ in terms of etonogestrel.

c Postpartum depression rank 726^th^ in terms of drospirenone.

## 4 DISCUSSION

The effects of many drugs, including hormonal contraceptive implants, on mental health are not completely understood. No study has yet focused on the effects of various contraceptive and therapeutic agents on PPD by using an SRS such as the FAERS database. In this study, we suggest that IUDs, hormonal contraceptives and SSRIs might be associated with an increased risk for PPD. The lower limit of the 95% CI for the RORs of levonorgestrel, etonogestrel, sertraline, drospirenone, fluoxetine, topiramate and quetiapine exceeds 1. Our findings point to the importance of drug safety evaluation by using real‐world data. The suggestion that PPD could be induced by an IUD is particularly important. Our results give essential knowledge to improve our understanding of this issue. This information may be particularly beneficial to nurses so that they can provide the best possible counselling to women.

Contraception is of great importance for women's reproductive life from both a social‐ and a health‐related standpoint (Toffol, Heikinheimo, Koponen, Luoto, & Partonen, 2011). Contraceptives such as levonorgestrel, etonogestrel and drospirenone are widely prescribed and are well tolerated. They have a well‐described side effect profile overall; however, information concerning the possible side effects of the contraceptives on mental health is relatively scant. Levonorgestrel, an IUD with progestogen, is a safe and effective device for contraception and the treatment of heavy menstrual bleeding (Hardeman & Weiss, 2014). Two levonorgestrel‐releasing IUDs are available in the USA: Mirena^®^ (Bayer HealthCare Pharmaceuticals Inc.), which releases 20 mg per day and Skyla^®^ (Bayer HealthCare Pharmaceuticals Inc.), which releases 14 mg per day (Wayne, 2013). Side effects of the levonorgestrel‐releasing IUD are similar to those of other progestin‐based contraceptives and include bleeding problems and hormonal adverse effects (Hardeman & Weiss, 2014; U. S. Food and Drug Administration, 2008; Wayne, 2013). Mild and rare hormonal side effects have been reported, such as mood changes, nervousness, nausea and headache (Luukkainen et al., 2001). The main concerns with levonorgestrel are IUD expulsion, dislocation and vaginal haemorrhage; for an etonogestrel device, the main problems are difficulty of use, product quality issues, pulmonary embolism and deep vein thrombosis; and for drospirenone, the main problems are pain, injury, pulmonary embolism, deep vein thrombosis and anxiety. Pulmonary embolism, deep vein thrombosis and vaginal haemorrhage might cause PPD.

Etonogestrel is a hormonal contraceptive implant (Nexplanon^®^, Merck & Co., Inc.). Side effects of Nexplanon^®^ include vaginal bleeding, vaginal infection, weakness and depression. More generally, depression is often mentioned as a possible side effect of contraceptive implants and women continue to report depression and other mood changes (Oinonen & Mazmanian, 2002). Mood changes, specifically depression, are among the most common reasons cited for discontinuing oral contraceptives (Oinonen & Mazmanian, 2002).

Drospirenone is an oral contraceptive, which is used safely in women with a range of medical conditions, including well‐controlled hypertension, uncomplicated diabetes mellitus and depression. In prospective studies, increased anxiety and increased depressive mood were reported in 7% and 10% of women on oral contraceptives respectively (Ernst, Baumgartner, Bauer, & Janssen, 2002).

In our study, the contraceptives levonorgestrel, etonogestrel and drospirenone showed a possible PPD signal (Table 3). Considering these data, we speculate that there may be an association between contraceptive use and PPD. The literature contains inconsistent results about the association between hormonal birth control and mental health. Several studies have shown less severe depressive symptoms and better overall physical function with the use of oral contraceptives (Young et al., 2007). Recent studies have indicated no significant association between contraceptives and depression (Duke et al., 2007; O'Connell et al., 2007). However, relationships between reductions in the concentrations of neuroactive steroids by oral contraceptives and mood or anxiety symptoms in healthy women have not been elucidated (Rapkin, Morgan, Sogliano, Biggio, & Concas, 2006).

The mechanism by which hormonal contraceptives may influence PPD remains unclear. One plausible reason for PPD caused by contraceptives might be adverse hormonal effects. Effects of sex steroids on the central nervous system have been widely characterized (Pluchino et al., 2009). Estrogens are known to regulate many neurotransmitter systems (serotonin, dopamine and noradrenalin) and progestins regulate serotonergic, opioidergic and cholinergic systems. For this reason, it is possible that there are neuropsychological effects associated with the use of contraceptives.

PPD is an area that should be further studied, as it is associated with older maternal age (Matsumoto et al., 2011) and primiparity. Evidence shows that primiparas have a significantly higher Edinburgh Postnatal Depression Scale (EPDS) score than the multiparas in late PPD. These characteristics of older postpartum primiparas also mean that they are a clinical concern from a nursing perspective. Information about contraceptives should be provided to postpartum women, particularly older postpartum primiparas. A summary of drugs associated with PPD among women of different ages is shown in Table 2. This research is also important for the care of postpartum women suffering autoimmune diseases. As shown in Table 1, adalimumab was mostly used for autoimmune diseases, such as rheumatoid arthritis, Crohn's disease, psoriasis and ulcerative colitis. Multiple sclerosis accounted for 71% of interferon beta‐1a cases. Women may need individualized support that would reduce their anticipated difficulty in adapting to hormonal contraceptives. The findings of this study can contribute to providing this individualized care.

Kennerley et al. showed that mothers experience maternity blues in the first few days after childbirth (Kennerley & Gath, 1989). Pitt reported that 50% of mothers suffered a spell of tearfulness and depression (Pitt, 1973). Stein showed that depression showed a peak around days 4–6 (Stein, 1980). However, the usage of contraceptives was not evaluated in these reports. The first postpartum month is considered to be the most difficult and problematic period for many mothers, with poor sleep quality and feelings of despair (Carolan, 2005; Iwata et al., 2015). Therefore, we considered a study of PPD during the first postpartum month to be important. However, women do not usually use contraceptives immediately after delivery. Because the date of childbirth is not listed in the FAERS database, we could not evaluate the duration from childbirth. Persistent PPD can occur at any time during the first year after delivery and lasts for a few weeks or months (O'Hara & Swain, 1996). The PPD associated with contraceptives and IUD in our results might be different from the maternity blues that often occur shortly postpartum as reported by Kennerley, Pitt or Stein.

PPD was observed in approximately 10–19% of Japanese women (Tamaki, 2008), which is similar to that observed in Western countries (Dennis & Creedy, 2004; World Health Organization, 2008). However, no reports were available from Japan in the FAERS database during the time period considered in our study. This might reflect differences in the frequency of drug usage, especially use of contraceptive devices and implants, between the USA and Japan.

Sertraline and fluoxetine are an antidepressant SSRIs. Quetiapine is an atypical antipsychotic for the treatment of schizophrenia and bipolar disorder, which is also concomitantly used with antidepressants to treat depression. Topiramate is an anticonvulsant drug. In our study, RORs with high values were observed for sertraline, fluoxetine, quetiapine and topiramate. We do not have a conclusive explanation for these data. However, associations of these drugs with depression have been reported at low frequencies, as stated on the package insert of the drug. We postulated that these adverse effects, although small, could partially explain the high values of the RORs in our study.

Several pharmacovigilance indexes have been developed to detect drug‐associated adverse events in SRSs, including the ROR, proportional reporting ratios (PRR), information component and empirical Bayes geometric mean, which are all widely used by regulatory authorities. Regulatory authorities may take regulatory action(s) to improve product safety and protect the public health based on these data. The PRR is currently used by the Medicines and Healthcare Products Regulatory Agency in the United Kingdom (UK), the ROR is used by the Pharmaceuticals and Medical Devices Agency in Japan and the Netherlands Pharmacovigilance Centre, the information component is used by the World Health Organization (WHO) and the empirical Bayes geometric mean is used by the FDA (Bates & Evans, 2009; Poluzzi, Raschi, Piccinni, & De Ponti, 2012). Although research on the performance, accuracy and reliability of different data mining algorithms is in progress, there is no recognized *gold standard* methodology.

Pharmacovigilance indexes, such as the ROR, have been developed to detect drug‐associated adverse events in the SRS, but these cannot be applied to evaluate the comparative strength of causalities (Bates & Evans, 2009). The ROR is a clear and easily applicable technique that allows for the control of covariates through logistic regression analysis (Abe et al., 2015; Suzuki et al., 2015; Ueda et al., 2015; Umetsu et al., 2015). An additional advantage of using the ROR is that non‐selective underreporting of a drug or adverse events has no influence on the value of the ROR compared with the population of patients experiencing an adverse event (Van der Heijden, van Puijenbroek, van Buuren, & van der Hofstede, 2002).

SRS is one of the primary tools used in pharmacovigilance because they reflect the realities of clinical practice (Poluzzi et al., 2012). With larger numbers of faithful reports, the FAERS database should help for optimizing pharmacotherapy (Sasaoka et al., 2016). Furthermore, Concato et al. reported that the results of well‐designed observational studies do not systematically overestimate the magnitude of the effects of treatment as compared with those in randomized, controlled trials on the same topic (Concato, Shah, & Horwitz, 2000). To the best of our knowledge, this is the first study to evaluate the potential risk of contraceptives on PPD in a real‐life setting. We hope these data will be the body of information that can be used by nurses to improve the management of women during the early postpartum period.

## 5 LIMITATIONS

We should note several limitations of our study. The definition of PPD is a subject of discussion. According to the Introductory Guide MedDRA Version 19.0, the PT is a distinct descriptor (single medical concept) for a symptom, sign, disease, diagnosis, therapeutic indication, investigation, surgical, or medical procedure and medical, social, or family history characteristic (International Council for Harmonisation, 2016). PTs should be unambiguous and as specific and self‐descriptive as possible in the context of international requirements. Therefore, eponymous terms are only used when they are recognized internationally. The granularity/specificity of the PT level is such that clinical pathologic or etiologic qualifiers of the descriptors are represented at the PT level (http://www.meddra.org/sites/default/files/guidance/file/intguide_19_0_english.pdf). It is difficult to confirm the criteria used to define PPD events by volunteers at the time of reporting. They only reported adverse events were according to ICH E2B, the international safety reporting guidelines and relied on the definitions provided by MedDRA.

The second limitation is a robustness of disproportionality analysis. The ROR is an index for the detection of an adverse event signal; however, it should be considered as exploratory in a context of signal detection. The ROR does not give sufficient evidence on causality and only offers a rough indication of signal strength. We should note that our study lacked detailed patient information related to possible confounding factors, such as psychological stress and obstetric/paediatrics factors, unwanted pregnancy and primiparity. As these factors are important in mood change and depression, robust epidemiological studies are recommended.

A third limitation is the timing of contraceptive administration, e.g., whether it was at the point of PPD or before pregnancy, is an important factor for the interpretation of our results. If it was before pregnancy it might not affect biological mechanisms at play after delivery and would therefore be irrelevant. In the FAERS database, administration data and the onset date of adverse events for each drug are not coded (U. S. Food and Drug Administration, “ASC_NTS.DOC”). There is no one‐to‐one correspondence between a drug and an adverse event. Therefore, we could not evaluate the precise timing of the administration of contraceptives. On the other hand, reports in the FAERS database have been reported by healthcare professionals. In our analysis, only reports with the drug code of “primary suspect” drug (PS) were included. We consider that the association of PPD and contraceptive drugs is therefore suggested by our data.

The FAERS database is a computerized spontaneous reporting system to which healthcare professionals and consumers send adverse event reports voluntarily through the MedWatch program (http://www.fda.gov/Safety/MedWatch/, cited 12 Jan 2017). To date, the FDA has only accepted electronic submissions of Individual Case Safety Reports in the XML format, prepared in accordance with ICH E2B to transmit information directly from database to database using standardized data elements. According to the “Instructions for Completing Form FDA 3500,” FDA staff knows the name, mailing address, phone number and E‐mail address of the person who can be contacted to give information on the event if follow‐up is necessary (http://www.fda.gov/Safety/MedWatch/HowToReport/DownloadForms/ucm149236.htm, cited 17 October 2016). Despite their best efforts, the FAERS database does not always contain enough patient background information to properly evaluate an event. In this study, we have not adjusted data for different regulatory activities. It could be possible by the logistic regression analysis, if we included the terms for various reporting nations, such as the USA and Japan among others, in the logistic model. The covariates should be evaluated with respect to a variety of patients’ backgrounds by using well‐organized epidemiologic studies in the future.

According to the DSM‐5, onset of PPD can begin at 4 weeks after delivery (American Psychiatric Association, 1998). Contraception is important for women who are postpartum, including those who are breastfeeding (Tepper, Phillips, Kapp, Gaffield, & Curtis, 2015; World Health Organization, 2010). As combined hormonal contraceptive may adversely affect milk production, these methods are generally not recommended for use during breastfeeding until 6 months postpartum. However, combined hormonal methods are an important contraceptive option during the postpartum period for non‐breastfeeding women (World Health Organization, 2010). We need to carefully consider the timing of contraceptive administration.

Initiation of contraception during the postpartum period is important to prevent unintended pregnancy and short birth intervals (World Health Organization, 2006). Hormonal contraceptives are prominent among contraceptive options. As the benefit and tolerability of contraceptives have been accepted worldwide, our results do not provide any justification for the restriction of contraceptive usage.

## 6 CONCLUSION

Among the drugs in the FAERS database, the use of contraceptives or an IUD with progestogen might pose a risk for PPD. We showed the potential risk of contraceptives on PPD in a real‐life setting. These data will enhance the information available to nurses and clinicians in advising patients on contraception and/or treating PPD and may be useful in the management of women's health during the early postpartum period. Considering the causality restraints of the current analysis, further epidemiological studies are recommended.

## 7 ETHICAL CONSIDERATIONS

Data from the FAERS database were obtained from the FDA website (www.fda.gov). Research ethics committee approval was not required for this study.

## CONFLICT OF INTEREST

JA is an employee of Medical Database. The rest of the authors have no conflict of interest.

## AUTHOR CONTRIBUTIONS

All authors have agreed on the final version and met at least one of the following criteria [recommended by the ICMJE (http://www.icmje.org/recommendations/)]:

- substantial contributions to conception and design, acquisition of data, or analysis and interpretation of data;
- drafting the article or revising it critically for important intellectual content.
